# Variation in Surgeon Proficiency Scores and Association With Digit Replantation Outcomes

**DOI:** 10.1001/jamanetworkopen.2021.28765

**Published:** 2021-10-26

**Authors:** Alfred P. Yoon, Robert L. Kane, Leyi Wang, Lu Wang, Kevin C. Chung

**Affiliations:** 1 Section of Plastic Surgery, Department of Surgery, University of Michigan Medical School, Ann Arbor; 2 Department of Biostatistics, University of Michigan School of Public Health, Ann Arbor

## Abstract

**Importance:**

Risk-adjusted variation in surgeon outcomes has been traditionally explained by surgeon volume and hospital infrastructure, yet it is unclear how a surgeon’s operative proficiency directly contributes to their patients’ outcomes.

**Objective:**

To assess the variation of surgeons’ operative proficiency and investigate its association with surgical outcomes.

**Design, Setting, and Participants:**

This case series was a retrospective analysis of all digit replantations and revascularizations at a single US university medical center between January 2000 and August 2020. Surgeons were assigned a proficiency score based on the expected procedure difficulty and outcomes from a sample of their cases. Surgeon proficiency scores were then used to determine associations with outcomes from subsequent cases. The expected difficulty of each case was calculated using a novel scoring system that applied pooled relative risks from a meta-analysis of risk factors for replantation and revascularization failure.

**Exposures:**

Digit replantation and revascularization.

**Main Outcomes and Measures:**

Digit survival at 1-month follow up (case success) and number of complications.

**Results:**

A total of 145 patients and 226 digits were treated by 11 surgeons with training in hand or microsurgery (mean [SD] age, 41.9 [15.2] years; 204 [90%] men); there were 116 replantations and 110 revascularizations. Surgeon proficiency scores ranged from 1.3 to 5.7, with a mean (SD) of 3.4 (1.4). Case success rates among surgeons varied from 20.0% to 90.5%, with a mean (SD) of 64.9%. Higher proficiency scores were associated with fewer case failures: each point increase was associated with 40% decreased odds of failure (odds ratio, 0.60; 95% CI, 0.38-0.94). Every 3-point increase in proficiency score was associated with 1 less complication (effect estimate, −0.29; 95% CI, −0.56 to 0.02). Surgeon proficiency score had a greater association with case failure than surgeon volume (16.7% vs 12.0%). The final model’s association with case failure had an area under the receiver operating characteristics curve of 0.93.

**Conclusions and Relevance:**

Operative proficiency varied widely among practicing surgeons and accounted for 17% of estimative ability for success of digit replantation and revascularization. Greater surgeon proficiency was associated with better outcomes, indicating that the value of surgical care may be optimized by improving surgeon proficiency.

## Key Points

**Question:**

Is surgeon proficiency associated with outcomes for digit replantation and revascularization?

**Findings:**

In this case series, greater surgeon proficiency, measured by a novel grading system for procedure difficulty that scored surgeon outcomes in digit replantation and revascularization, was associated with a greater likelihood of digit survival and fewer complications even after controlling for case mix and injury characteristics known to affect outcomes. Surgeon proficiency scores accounted for 17% of the variation in digit survival.

**Meaning:**

These results suggest that improving surgeon proficiency may not only improve quality of surgical care delivered but also optimize health care resource use.

## Teaser

This case series examines the association of a novel grading system for surgeon proficiency in digit replantation and revascularization procedures with patient outcomes.

## Introduction

Anders Ericsson^1^ suggested that innate differences in human ability can be overcome through 10 000 hours of deliberate practice. This notion that “practice makes perfect” is central to surgical training programs. Differences in operative skill among new surgical interns are presumed to equalize after a minimum number of cases, producing surgeons with comparable levels of ability.^2^ Nevertheless, evidence shows there is significant variation in risk-adjusted outcomes among attending surgeons in various procedures.^3,4,5,6,7^ To explain this phenomenon, many studies have focused on the positive correlation between a surgeon’s procedure volume and their outcomes. However, these investigations have largely ignored whether a surgeon’s operative proficiency could directly contribute to the equation. A link between surgeon ability and patient outcomes is logical, yet few studies have strived to measure the operative proficiency of attending surgeons and determine its association with outcomes.

Several investigators estimated operative proficiency by assessing videos of surgeons’ operations and grading their technical skills.^8,9,10^ In these studies of bariatric surgeons, technical skill varied widely, accounting for 26% of the variation in postoperative complications,^9^ and had the greatest association with outcomes in complex procedures.^11^ Although video-based evaluations are practical for bariatric surgery, this approach is not suitable for measuring operative proficiency for emergent procedures. Furthermore, it is unclear whether an association between surgeon proficiency and outcome exists in highly complex surgeries such as digit replantation and revascularization. The average success rate of digit replantation at tertiary academic centers can be as low as 57%.^12^ Failed replantation not only detrimentally affects patient quality-of-life, but also incurs considerable financial waste; the total direct and indirect cost of a single digit replantation is approximately $15 000.^13^ Identifying the association among operative proficiency and outcomes for digit replantation and revascularization may have implications for optimizing national referral patterns and setting competency benchmarks to improve the value of care.

We conducted a retrospective medical record review at a US university medical center to investigate the association between surgeons’ operative proficiency and outcomes for digit replantation and revascularization. Surgeon proficiency scores were calculated using a novel evidence-based scoring system that graded the difficulty of each replantation and revascularization performed. We hypothesized that the operative proficiency score would explain a significant percentage of variability in outcomes after case mix adjustment, and that higher proficiency scores would be associated with lower complication rates.

## Methods

### Study Design

We conducted a retrospective case series study of patients with traumatic digit amputations who underwent replantation or revascularization at Michigan Medicine from January 1, 2000, to August 31, 2020. We used *Current Procedural Terminology* (CPT) and *International Classification of Disease, Ninth Revision* (*ICD-9*) codes to identify and confirm our study group from the electronic medical record (EMR) (eTable 1 in the Supplement). Patients older than age 18 years with at least 1 month of follow-up were included. We excluded digits that were converted to revision amputation intra-operatively and digits that were revascularized without preoperative stigmata of digital ischemia. If a patient sustained traumatic amputation of multiple digits, we only included digits that were amenable to replantation at the time of injury as documented in the operative report by the surgeon. The study was conducted between April 1, 2020, and December 15, 2020; data analysis was conducted between December 16, 2020, and February 7, 2021. We adhered to the uncontrolled case series reporting guideline for case series published by Kempen.^14^ This study was considered exempt from regulation and informed consent requirements by the University of Michigan institutional review board because of anonymized secondary use of identifiable data.

### Surgeon, Patient, and Injury Variables and Outcomes

Based on published risk factors for replantation failure,^12,15,16,17,18,19,20,21,22,23,24,25,26,27,28,29,30,31,32,33,34,35,36,37,38,39,40,41,42,43,44^ the following patient and injury characteristics were collected from the EMR: case type (replantation or revascularization), patient age, sex, smoking status, Elixhauser score, affected digit, mechanism of injury, ischemia time, zone of injury, number of anastomosed arteries and veins, and use of vein graft. Replantation was defined as the reattachment of a completely amputated digit (including bone, tendon, neurovascular structures, and skin) that required anastomosis of both artery and vein. Revascularization was defined as the repair of a partially amputated digit with an intact skin bridge and clinical signs of neurovascular compromise. For each surgeon, the following variables were calculated: total replantation and revascularization cases, years of practice, and surgeon proficiency score. The primary outcome of this study was case success, defined as a viable replanted or revascularized digit at 1-month follow-up visit. Typically, necrosis of the reconstructed digit will be apparent at 1 week after surgery. The secondary outcome was total complications, including stiffness, nonunion, severe infections, and any associated revision surgeries.

### Procedure Difficulty Score

We developed an evidence-based scoring system to grade the procedure difficulty of digit replantations and revascularizations. A literature search identified 31 outcome studies that reported patient and perioperative factors associated with digit survival following replantation and revascularization.^12,15,16,17,18,19,20,21,22,23,24,25,26,27,28,29,30,31,32,33,34,35,36,37,38,39,40,41,42,43,44^ We used these studies to compile relative risk data for the following factors associated with case failure: complete amputation as opposed to partial amputation, active smoking status, multidigit injury, crush or avulsion injury, distal amputations, and fewer arterial or venous anastomoses performed (Table 1; see eTable 2 in the Supplement for the complete set of data that were used to calculate the pooled relative risk for each variable). A fixed-effect meta-regression model was fit to calculate the pooled relative risk of replantation or revascularization failure for each patient and injury characteristic reported in the literature and to identify statistically significant variables associated with case success. The procedure difficulty score was calculated by multiplying the pooled relative risk of replantation or revascularization failure that was associated with each risk factor present in a case. For example, the procedure difficulty score for treating a current smoker with complete digit amputation following an avulsion injury was: baseline pooled relative risk of replantation failure × pooled relative risk of failure in current smokers × pooled relative risk of failure for avulsion amputations.

**Table 1.** Pooled Relative Risk of Covariates Associated With Failure of Digit Replantation and Revascularization

Covariates	Pooled relative risk (95% CI)^a^	*P* value
Procedure (replantation vs revascularization)	1.52 (1.11-2.09)	.009
Smoking status (smoker vs nonsmoker)	2.31 (1.83-2.92)	<.001
No. of digits (multiple vs single digit)	1.79 (1.57-2.04)	<.001
Mechanism of injury (crush vs sharp)	2.04 (1.43-2.92)	<.001
Mechanism of injury (avulsion vs sharp)	2.52 (1.79-3.54)	<.001
Zone of injury (Tamai zones 1 and 2 vs others)	1.31 (1.04-1.65)	.02

^a^ The pooled relative risk for each patient and injury characteristics was calculated using a fixed effect meta-regression model.

### Surgeon Proficiency Score

Surgeons’ cases were sorted chronologically and divided into 2 halves with an equal number of cases. Cases from the first half were used to calculate a surgeon’s proficiency score, which was then used to determine associations with the primary and secondary outcomes in the second half of cases. This methodology was selected over dividing each surgeon’s series based on number of years to ensure balanced case numbers in both data sets. Surgeon proficiency score was determined by adjusting the surgeon’s overall success rate by each procedure’s difficulty score using the following equation:


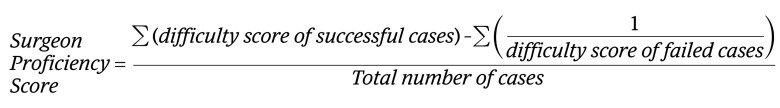


The first term of the numerator sums all the difficulty scores of a surgeon’s successful cases, rewarding the surgeon with more points when successful cases were more difficult. The second term in the formula is the sum of the inverse of each difficulty score from all failed cases. Subtracting the second term from the first was intended to penalize surgeons more heavily when they failed a less challenging case. The numerator was divided by the total number of cases to prevent overinflation of scores by procedure volume. Volume was adjusted as a separate variable in the regression model.

### Statistical Analysis

Two outcomes were considered: case success and total complications per digit. Associations between proficiency score and case success were investigated with a mixed-effect logistic regression model using the second half of surgeons’ cases using odds ratios (ORs). Surgeon proficiency score was retained in the model as a continuous variable. Similarly, the association between proficiency score and total complications per digit was evaluated through mixed-effects linear regression models. The unit of analysis was each digit because a patient could have sustained multiple digit amputations and each digit may have had different complications and outcomes. In all models, random effects were introduced to account for clustering of patients by surgeons. A receiver operating characteristic curve of the mixed-effect logistic regression model was plotted and the area under the curve (AUC) was used to evaluate its accuracy for identifying replantation success. The ability of the model to determine associations with case success rates and procedure difficulty, surgeon proficiency, and procedure volume in terms of AUC was compared and reported. All covariates in the study were also compared between successful and failed cases using *t* tests for continuous variables and a Pearson χ^2^ test or Fisher Exact test for categorical variables. The following patient-level covariates were adjusted in the model: age, sex, Elixhauser comorbidity score, number of venous anastomoses, number of arterial anastomoses, presence of vein graft, length of hospital stay, and procedure difficulty score. Surgeon covariates included years of experience, total volume of digit replantations and revascularizations, and proficiency score. Collinearity among surgeon proficiency score (based on the first half of surgeon case series), surgeon volume (based on the first half of surgeon case series), and procedure difficulty score (based on the second half of surgeon case series) were assessed. Spearman correlation between surgeon proficiency score and procedure volume was 0.37 (*P* < .001) and that of surgeon proficiency score and procedure difficulty score was 0.11 (*P* = 0.27). An a priori significance level was set at *P* < .05 in 2-sided tests. All analyses were performed using R version 3.6.2 and R Studio version 1.3.959 (R Foundation for Statistical Computing).

## Results

A total of 145 patients and 226 digits met inclusion criteria, of which 51% (116) underwent replantation and 49% (110) underwent revascularization (Table 2). Of the total patient cohort, 90% (204) were men and 10% (22) were women, with a mean (SD) age of 41.9 (15.2) years. The study cohort was formed by 11 surgeons who participated in replantation call. The surgeons had a range of 3 to 16 years of independent surgical experience at the start of the assessment period (median [IQR] experience, 8 [7.3] years). Nine surgeons were hand-fellowship trained, and 2 surgeons were microsurgery-fellowship trained. Surgeons varied in the total number of cases performed, with a mean (SD) of 21 (12) cases and a range of 9 to 44 cases. Among 226 included digits, 155 digits (68.9%) were successfully replanted or revascularized.

**Table 2.** Descriptive Statistics of Replanted and Revascularized Digits

Characteristics	Procedures, No. (%)			*P* value^c^
	All digits (n = 226)	Failed digits (n = 71)^a^	Successful digits (n = 155)^b^	
Age, mean (SD)	41.9 (15.2)	42.4 (15.2)	42.7 (15.7)	.73
Sex				
Men	204 (90)	61 (86)	143 (92)	.14
Women	22 (10)	10 (14)	12 (8)	
Smoking status				
Current	89 (39)	30 (42)	59 (38)	.23
Former	26 (12)	8 (11)	18 (12)	
Never	97 (43)	32 (45)	65 (42)	
Unknown	14 (6)	1 (1)	13 (8)	
Elixhauser score				
0	116 (51)	29 (41)	87 (56)	<.001
1	61 (27)	26 (37)	35 (23)	
2	21 (9)	5 (7)	16 (10)	
3	13 (6)	2 (3)	11 (7)	
4	8 (4)	3 (4)	5 (3)	
≥5	7 (3)	6 (8)	1 (1)	
Multidigit amputation				
Yes	132 (58)	44 (62)	88 (57)	.46
No	94 (42)	27 (38)	67 (43)	
Mechanism of injury				
Sharp	96 (42)	27 (38)	69 (45)	.16
Crush	75 (33)	21 (30)	54 (35)	
Avulsion	55 (24)	23 (32)	32 (21)	
Procedure				
Replantation	116 (51)	55 (77)	61 (39)	<.001
Revascularization	110 (49)	16 (23)	94 (61)	
Ischemia time, h				
<12	203 (90)	66 (93)	137 (88)	.35
≥12	23 (10)	5 (7)	18 (12)	
Zone of injury				
Zone 1	6 (3)	6 (8)	0	.002
Zone 2	134 (59)	32 (45)	102 (66)	
Zone 3	27 (12)	9 (13)	18 (12)	
T1	19 (8)	8 (11)	11 (7)	
T2	34 (15)	13 (18)	21 (14)	
T3	5 (2)	3 (4)	2 (1)	
T4	1 (0)	0	1 (1)	
Affected digit				
Small	18 (8)	6 (8)	12 (8)	.44
Ring	45 (20)	12 (17)	33 (21)	
Middle	60 (27)	18 (25)	42 (27)	
Index	44 (19)	11 (15)	33 (21)	
Thumb	59 (26)	24 (34)	35 (23)	
No. of digital arteries anastomosed				
Unknown	4 (2)	4 (6)	0	.02
1	177 (78)	54 (76)	123 (79)	
2	45 (20)	13 (18)	32 (21)	
No. of digital veins anastomosed				
NA^d^	85 (38)	11 (15)	74 (48)	<.001
0	23 (10)	13 (18)	10 (6)	
1	80 (35)	32 (45)	48 (31)	
2	34 (15)	13 (18)	21 (14)	
3	4 (2)	2 (3)	2 (1)	
Vein graft				
Yes	55 (24)	22 (31)	33 (21)	.11
No	171 (76)	49 (69)	122 (79)	

Abbreviation: NA, not applicable.

^a^ Failed digits defined as replanted or revascularized digits that were not viable at 1 month follow-up.

^b^ Successful digits defined as replanted or revascularized digits that were viable at 1 month follow-up.

^c^ Differences in means for continuous variables were assessed using *t* tests; categorical variables were assessed using χ^2^ tests or Fisher exact test.

^d^ Skin bridge intact, obviating the need for venous anastomosis.

Among the 11 surgeons included in this study, the case success rates in the latter half of surgeons’ careers ranged from 20.0% to 90.5%, averaging 64.9%. Surgeon proficiency scores also varied widely from 1.3 to 5.7, with a mean (SD) of 3.4 (Table 3). The proficiency score demonstrated adequate variation to enable differentiation among surgeons, as the range (1.26-5.71, difference = 4.45) was 3 times wider than the SD. Surgeon proficiency score and success rates from the second half of cases were highly correlated, with a correlation coefficient of 0.70 (eFigure in the Supplement).

**Table 3.** Surgeon Proficiency Scores

Surgeon	First half of case series^a^					Second half of case series^b^				
	Surgeon proficiency score	Failed cases (n = 33), No.	Total case volume (n = 115), No.	Success rate, %	Average procedure difficulty	Surgeon proficiency score	Failed cases (n = 39), No.	Total case volume (n = 111), No.	Success rate, %	Average procedure difficulty
A	4.0	3	22	86.4	5.6	1.8	9	22	59.1	4.3
B	4.2	7	22	68.2	6.2	3.6	2	21	90.5	4.1
C	4.7	1	7	85.7	5.4	2.8	1	6	83.3	4.3
D	1.5	6	12	50.0	8.1	1.1	8	10	20.0	4.3
E	1.9	2	6	66.7	2.7	0.6	3	5	40.0	7.6
F	3.6	0	5	100.0	3.6	1.3	2	4	50.0	4.5
G	1.3	3	6	50.0	2.6	1.0	3	8	62.5	2.7
H	1.9	4	6	33.3	6.3	0.6	5	7	28.6	4.5
I	5.7	1	7	85.7	6.2	2.7	2	6	66.7	4.1
J	4.8	2	7	71.4	5.9	3.6	2	8	75.0	6.8
K	3.9	4	15	73.3	4.9	2.6	2	14	85.7	3.4
Mean (SD)	3.4 (1.5)	3 (2.1)	11 (6.4)	71.3 (19.6)	5.2 (1.7)	2.0 (1.1)	4 (2.7)	10 (6.3)	64.9 (23.4)	4.6 (1.4)

^a^ Calculated from first half of surgeons’ cases. Failed cases are replanted or revascularized digits that were not viable at 1 month follow-up. Total case volume is the sum of all digit replantations and revascularizations performed by the surgeon. Average procedure difficulty is the mean procedure difficulty from all digit replantations and revascularizations performed by the surgeon in the specified time frame.

^b^ Calculated from second half of surgeons’ cases. Success rate refers to the percentage of all digit replantations and revascularizations performed by the surgeon that were viable at 1 month follow-up.

The regression model was highly accurate in identifying case success, with an AUC of 0.93. A 1-point increase in procedure difficulty score was associated with a 63% increase in odds of case failure (OR, 1.63; 95% CI, 1.27-2.10), whereas a 1-point increase in surgeon proficiency score was associated with a 40% decrease in odds of failure (OR, 0.60; 95% CI, 0.38-0.94) (Table 4). The difference in case success and failure was explained to the greatest degree by procedure difficulty score (25.7%), followed by surgeon proficiency score (16.7%), and total surgeon volume (12.0%). Greater surgeon proficiency score was also associated with fewer complications; every 3-point increase was associated with 1 less total complication (effect estimate [EE], −0.29; 95% CI, −0.56 to −0.02). Each additional year of surgeon experience was associated with increased likelihood of case failure (EE, 1.15; 95% CI, 1.05 to 1.27).

**Table 4.** Multivariable Regression Results of Patient and Surgeon Characteristics

Characteristics^a^	Model 1: Case failure, OR (95% CI)	*P* value	Model 2: Complications, OR (95% CI)^b^	*P* value
Patient characteristics				
Age, y	1.00 (0.96 to 1.05)	.83	0.00 (−0.02 to 0.02)	.79
Men	0.19 (0.03 to 1.15)	.07	−0.30 (−1.40 to 0.81)	.60
Elixhauser score	1.07 (0.67 to 1.70)	.79	−0.06 (−0.29 to 0.17)	.62
No. of venous anastomoses	0.78 (0.50 to 1.21)	.27	−0.18 (−0.41 to 0.05)	.12
Presence of vein graft	1.23 (0.30 to 5.09)	.77	−0.51 (−1.28 to 0.26)	.20
Length of stay, d	1.02 (0.88 to 1.19)	.78	0.06 (−0.01 to 0.10)	.11
Procedure difficulty score	1.63 (1.27 to 2.10)	<.001	−0.01 (−0.11 to 0.10)	.87
Surgeon characteristics				
Experience, y	1.15 (1.05 to 1.27)	.004	0.06 (0.01 to 0.11)	.01
Procedure volume	0.91 (0.83 to 1.00)	.06	−0.04 (−0.09 to 0.01)	.12
Proficiency score	0.60 (0.38 to 0.94)	.03	−0.29 (−0.56 to −0.02)	.04

Abbreviation: OR, odds ratio.

^a^ ORs refer to 1 unit increases except for sex (men vs women) and presence of vein graft (yes vs no).

^b^ Complications included nonunion, revision surgery, stiffness, and severe infection.

## Discussion

We observed that operative proficiency in digit replantation and revascularization varied substantially among attending surgeons at a tertiary academic center, and that greater surgeon proficiency was associated with improved outcomes. For each point increase in a surgeon’s operative proficiency score, the odds of case failure decreased by approximately 40%. Overall complication rates for replantation and revascularization were also significantly lower for surgeons with higher proficiency scores. Our findings suggest that a surgeon’s operative proficiency, measured by a case mix–adjusted scoring system, can be a means to maximize patient outcomes. For complex procedures such as digit replantation, surgeon proficiency may have an independent and stronger association with outcomes when compared with procedure volume alone.

Many studies, including our own, support a volume-outcome relationship for surgeons, but few have quantified the association between surgeon proficiency on outcomes and presented this finding in parallel with surgeon volume. Stulberg et al^9^ used video-based evaluations to measure surgeons’ technical skills, reporting that 26% of variation in postcolectomy complications was attributed to surgeon skill. However, there was no mention of the proportion of outcome variation attributed to surgeon volume, making it difficult to compare the independent associations of surgeon performance and surgeon volume on patient outcomes. In our study, operative proficiency score explained the variation in outcomes to a greater degree than procedure volume. Furthermore, operative proficiency score, but not procedure volume, was associated with surgeons’ overall complication rates. This implies that surgeon volume and operative performance may have synergistic associations with some outcomes but distinct associations with others. It is plausible that some surgeons, despite having high procedural volume, are prone to repeating certain mistakes or improper techniques unless these are identified and corrected.

Previous attempts have been made to characterize the expertise of a surgeon without the use of intraoperative data such as video recordings. Tang and Giddins^45^ developed a framework for determining the expertise of surgeons based on years of experience and academic contributions related to the surgical procedure in question. The purpose of this framework was to improve the reporting of surgeon expertise in original research that presents outcomes from a group of surgeons who often have varying degrees of training and experience. However, the role of surgeon experience in relation to outcomes remains unclear, particularly for highly complex procedures. Surprisingly, our study found that years of practice was associated with higher failure rates, although this should be interpreted cautiously. This finding may indicate that more years of practice does not necessarily translate to increased surgeon skill. It is conceivable that for highly complex procedures, some surgeons with lower operative proficiency may continue to perpetuate poor operative techniques that may result in poor surgical outcomes. It is also important to note that some senior surgeons who have been in practice longer may not perform replantations as regularly as their more junior peers. Although Tang and Giddins’ methodology may be applicable to many surgical procedures, we aimed to develop a more objective measure of surgeon proficiency based on risk factors known to affect procedural difficulty and surgeon outcomes.

Surgeons with lower operative proficiency scores were more likely to incur greater hospital charges when compared with higher proficiency surgeons. These data indicate that surgeon performance can be leveraged to increase the value of surgical care. Such initiatives are important given the unsustainable trajectory of health care spending in the US.^46^ Owing to an established volume-outcome relationship in the literature, volume-based metrics have been a guiding force in efforts that promote value of care. For digit replantation, it has been shown that centralizing the procedure at high-volume centers with high-volume physicians could more than double the likelihood of successful outcomes in the US, thereby dramatically reducing costs.^47^ Although volume-based centralization of replantation could render considerable cost savings, our study suggests that data on surgeons’ operative performance provides another layer of insight for improving outcomes. Data on surgeons’ operative performance could therefore be of interest to a variety of stakeholders, including health care purchasers and hospital administrators. Private regulators have already implemented volume-based restrictions that determine which surgeons can perform high-risk procedures at designated centers of excellence.^48^ Some health care purchasers may refer subscribers to hospitals with favorable indicators of surgeon skill or operative performance. In other words, a more direct measure of surgeon proficiency beyond volume could be used by regulatory groups to ensure that only the highest-performing surgeons or surgical centers can offer certain complex procedures.

The findings from our study raise questions regarding what can be done to improve the proficiency of surgeons with low operative performance scores. Although practice is essential for improvements in performance, not all methods of practice are equivalent. To maximize performance increase from practice, 5 elements of deliberate practice have been described.^49,50,51^ The trainee must be motivated, receive clear learning objectives from an expert, practice relevant skills with focus, measure their performance, and receive immediate feedback from an expert. Therefore, surgeons with lower operative performance should not only practice the essential techniques necessary for the procedure, but also receive targeted feedback and engage in a highly structured training program. For example, an institution may identify surgeons with consistently poor operative performance in digit replantation and require them to practice achieving tension-free microvascular anastomosis through laboratory simulation exercises. Surgeons who are highly motivated to improve and diligently train in microvascular anastomoses in the lab with feedback from a dedicated mentor fulfill the essential components of deliberate practice (Figure), and would be expected to demonstrate improvements in their operative performance.^52^

**Figure.**
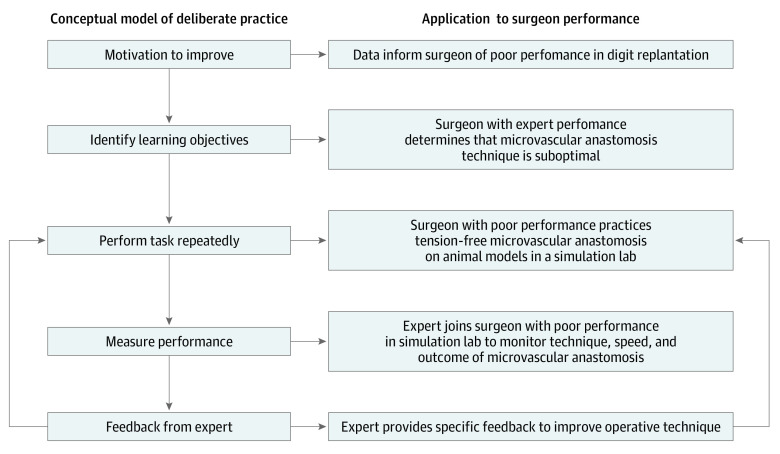
Study Flowchart

### Limitations

Our investigation had several limitations. First, we recognize that we did not directly measure surgeons’ technical skills, which would have required intraoperative data capture.^53^ Our investigation used retrospective outcomes data to estimate surgeon performance through an evidence-based operative proficiency score. However, because this score was derived from the end result of a surgeon’s care, we viewed it as encompassing a range of factors beyond just technical skill, including intraoperative judgement, surgical team dynamics, and perioperative decision-making, all of which can affect outcomes. Given that over 40% of surgical errors arise from communication failures,^54^ focusing on technical skill alone may not accurately capture a surgeon’s operative proficiency. Furthermore, for emergent procedures such as digit replantation, video recordings of the surgeon are impractical as these cases have unpredictable scheduling and often occur in the early hours of the morning. Another limitation was that this was a single-center study with a modest sample size. When selecting our study design, we chose to extract data from the EMR at our institution as opposed to an administrative data set, knowing that 1 tradeoff would be loss of sample size. However, the EMR offers a rich set of clinical variables and detailed operative notes, enabling a more in-depth analysis of case difficulty and capture of complications up to 6 months postoperatively. Lastly, the decline in surgeon proficiency score in the second half of their career compared with the first half was unexpected. However, based on both lower average procedure difficulty and average success rate of the second half, a lower surgeon proficiency score is mathematically sound. The reason behind this finding is unclear and is a topic of future research.

## Conclusions

Surgeon proficiency in digit replantation and revascularization varied widely at a single tertiary care institution. Surgeon proficiency scores were more strongly correlated with outcomes compared with surgeon volume and greater surgeon proficiency was associated with fewer complications including failure. These associations were present even after controlling for case mix, surgeon procedure volume, surgeon training, and years of experience. Efforts to improve surgeon proficiency in highly complex procedures can improve patient outcomes and yield health care savings.

## Notes

Supplement. **eTable 1.** Diagnosis and Procedure Codes of Interest **eTable 2.** Pooled Relative Risk of Covariates Associated with Failure of Digit Replantation and Revascularization **eFigure.** Correlation Between Surgeon Proficiency and Replantation/Revascularization Outcomes
