# Supplementary material for: Variation in Surgeon Proficiency Scores and Association With Digit Replantation Outcomes
Source: JAMA Netw Open. 2021 Oct 26;4(10):e2128765. doi: 10.1001/jamanetworkopen.2021.28765 (PMC8548947; doi:10.1001/jamanetworkopen.2021.28765)
Supplement: Supplement. — eTable 1. Diagnosis and Procedure Codes of Interest eTable 2. Pooled Relative Risk of Covariates Associated with Failure of Digit Replantation and Revascularization eFigure. Correlation Between Surgeon Proficiency and Replantation/Revascularization Outcomes [file jamanetwopen-e2128765-s001.pdf]

Supplemental Online Content

Yoon AP, Kane RL, Wang L, Wang L, Chung KC. Variation in Surgeon Proficiency Scores and Association With Digit Replantation Outcomes. *JAMA Netw Open.* 2021;4(10):e2128765. doi:10.1001/jamanetworkopen.2021.28765

eTable 1. Diagnosis and Procedure Codes of Interest

eTable 2. Pooled Relative Risk of Covariates Associated with Failure of Digit Replantation and Revascularization

eFigure. Correlation Between Surgeon Proficiency and Replantation/Revascularization Outcomes

This supplemental material has been provided by the authors to give readers additional information about their work.

© 2021 Yoon AP et al. *JAMA Network Open.*

eTable 1. Diagnosis and Procedure Codes of Interest

Coding System	Definition	Codes
ICD-9 Diagnosis Code	Thumb reattachment	84.21
	Finger reattachment	84.22
CPT Code	Replantation of thumb (complete)	20827, 20824
	Replantation of digit (complete)	20816, 20822
	Repair blood vessel, hand/finger	35207
	Phalangeal fracture fixation	26727, 26735
	Flexor tendon repair	26350, 26352, 26356, 26357, 26358, 26370, 26372, 26373
	Extensor tendon repair	26410, 26412, 26418, 26420, 26426
	Digital nerve repair	64702, 64704, 64831, 64832, 64872
	Myocutaneous, osteocutaneous, or fasciocutaneous free tissue transfer	15756, 15757, 15758, 20955, 20962, 20969, 20970

CPT: Current Procedural Terminology

ICD-9: International Classification of Disease, Ninth Revision, Clinical Modification

© 2021 Yoon AP et al. *JAMA Network Open.*

eTable 2: Pooled Relative Risk of Covariates Associated with Failure of Digit Replantation and Revascularization

Covariate A	Failed Cases Covariate A	Successful Cases Covariate A	Covariate B	Failed Cases Covariate B	Successful Cases Covariate B	Pooled Relative Risk of Covariate A‡	95% CI†	P-value
Procedure (replantation vs. revascularization)	176 / 1,069 (16%)	893 / 1,069 (84%)	Revascularization	40 / 384 (10%)	344 / 384 (90%)	1.52	(1.11, 2.09)	< 0.01*
Smoking status (smoker vs. nonsmoker)	69 / 162 (43%)	93 / 162 (57%)	Nonsmoker	93 / 772 (12%)	679 / 772 (88%)	2.31	(1.83, 2.92)	< 0.01*
Number of digits (multiple vs. single digit)	188 / 407 (46%)	219 / 407 (54%)	Single digit amputation	642 / 2,660 (24%)	2,018 / 2,660 (76%)	1.79	(1.57, 2.04)	< 0.01*
Mechanism of injury (crush vs. sharp)	102 / 944 (11%)	842 / 944 (89%)	Sharp injury	66 / 556 (12%)	490 / 556 (88%)	2.04	(1.43, 2.92)	< 0.01*
Mechanism of injury (avulsion vs. sharp)	64 / 253 (25%)	189 / 253 (75%)	Sharp injury	58 / 472 (12%)	414 / 472 (88%)	2.52	(1.79, 3.54)	< 0.01*
Zone of injury (Tamai zone 1&2 vs. others)	129 / 496 (26%)	367 / 496 (74%)	Other Tamai Zones	148 / 615 (24%)	467 / 615 (76%)	1.31	(1.04, 1.65)	0.02*

† 95% Confidence Interval

‡ The pooled relative risk for each patient and injury characteristic was calculated using a fixed effect meta-regression model

*Statistical significance

© 2021 Yoon AP et al. *JAMA Network Open.*

eFigure 1: Correlation Between Surgeon Proficiency and Replantation/Revascularization Outcomes

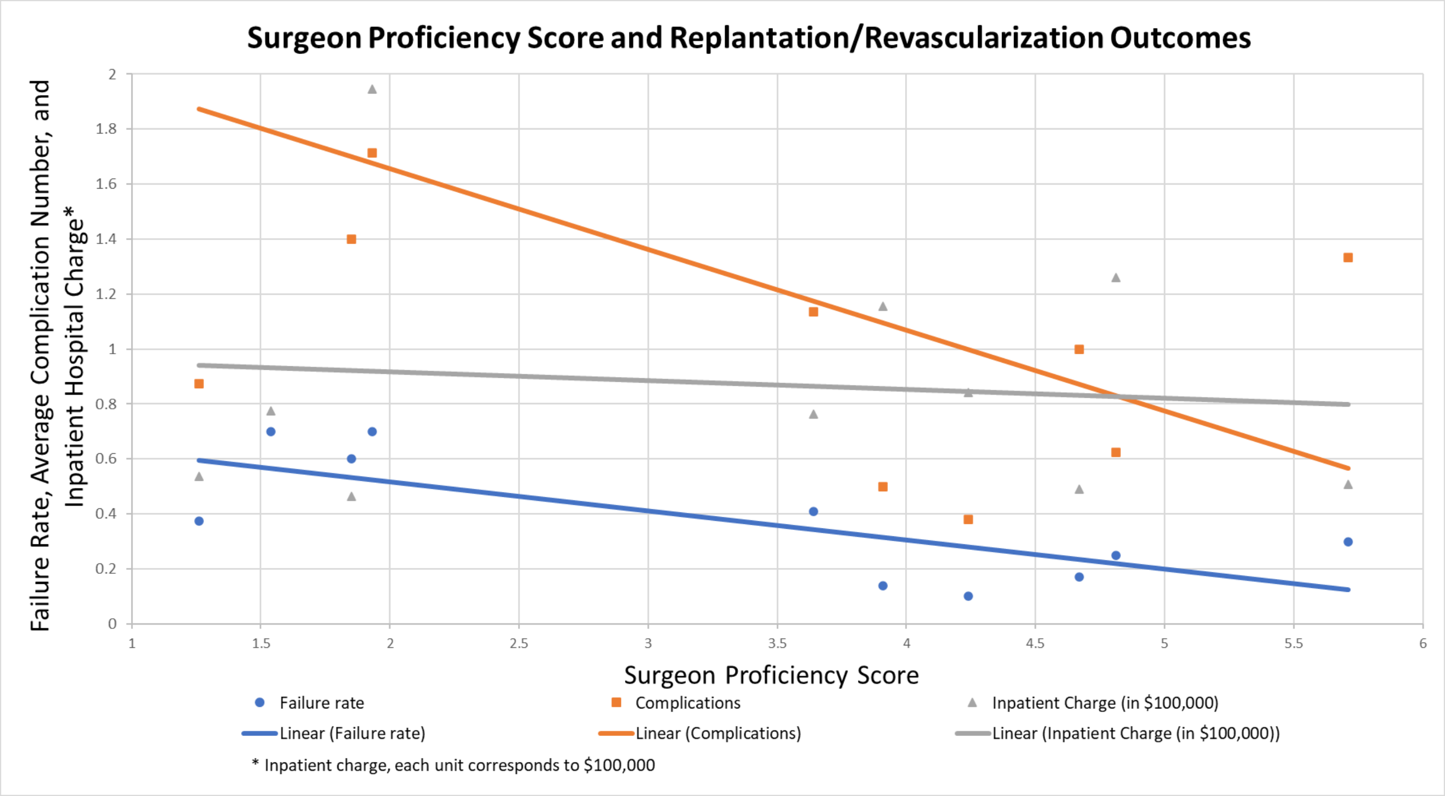

© 2021 Yoon AP et al. *JAMA Network Open.*
